# Disseminated Langerhans cell histiocytosis presenting as oesophageal disease in a cat

**DOI:** 10.1177/2055116919874902

**Published:** 2019-09-19

**Authors:** Edward Bellamy, Stefano Di Palma, Lorenzo Ressel, Elisabet Domínguez, Yordan Fernández

**Affiliations:** 1 Centre for Small Animal Studies, Animal Health Trust, Newmarket, UK; 2 Department of Pathology and Public Health, Institute of Veterinary Science, University of Liverpool, Neston, UK

**Keywords:** Langerhans cell, Birkbeck granules, histiocyte, oesophagus

## Abstract

**Case summary:**

An 11-year-old female spayed domestic shorthair cat was referred with a 2-month history of ptyalism, hyporexia and weight loss. Physical examination revealed reduced body condition score (2/9) and decreased skin turgor. Laboratory abnormalities included mild erythrocytosis, elevated creatine kinase, hypercobalaminaemia and hypofolataemia. CT of the head and abdominal ultrasonography were within normal limits. Gastroesophagoscopy revealed mucosal ulceration and possible stenosis of the distal oesophagus. Thoracic radiographs and iodine oesophagram showed a soft tissue opacity in the caudodorsal thorax compatible with a parietal oesophageal mass causing luminal stenosis or an extra-parietal mass causing ventral displacement and compression of the oesophagus. Pulmonary nodules were observed in the cranial lung lobes. CT of the thorax confirmed the oesophageal origin of the mass and the presence of pulmonary nodules scattered throughout the lung parenchyma. The patient was euthanased given the imaging findings and perceived guarded prognosis. Post-mortem examination revealed multifocal nodular lesions affecting the oesophagus, lungs, kidneys and pancreas. Histopathological examination identified atypical round cells characterised by eosinophilic cytoplasm and pale nuclei with prominent nuclear grooves, compatible with neoplastic histiocytic cells. Immunohistochemistry revealed strong expression for CD18, Iba-1 and vimentin. Transmission electron microscopy demonstrated intracytoplasmic organelles consistent with Birkbeck granules of Langerhans cell origin in lesional histiocytes. These findings were compatible with a diagnosis of disseminated Langerhans cell histiocytosis.

**Relevance and novel information:**

To our knowledge, this is the first report of disseminated Langerhans cell histiocytosis with oesophageal involvement in a cat.

## Introduction

Histiocytic proliferative diseases are derived from cells of macrophage or dendritic lineage.^1^ Dendritic cells (DCs), which include interstitial DC and Langerhans cells (LCs), differentiate from a common CD34^+^ stem cell precursor in bone marrow.^2,3^ The C-type lectin, langerin (CD207), is expressed on the surface of LCs.^4^ Internalisation of langerin mediates the formation of Birkbeck’s granules (BGs), which are cytoplasmic structures that define LCs and distinguish them from interstitial DCs.^4^ LCs colonise the epithelia of mucous membranes of the tongue, oropharynx, oesophagus, vagina, bronchi and epidermis.^5,6^ As sentinels of the immune system, DCs are responsible for antigen uptake, processing and presentation.^7^

LC proliferative disorders have been best described in humans.^8^ Human LC histiocytosis (LCH) covers a spectrum of disease, ranging from unifocal to rapidly progressive multifocal multisystem disease.^8^ Feline histiocytic disorders of LC origin are uncommon.^8^ Pulmonary LCH has been described in cats as pulmonary disease alone or with multi-organ involvement, including the lungs, pancreas, kidneys, spleen, liver, thyroid, parathyroid gland and tracheobronchial, hepatosplenic and mesenteric lymph nodes.^4,5,9^ A study describing the clinical, morphological and immunophenotypic features of feline progressive histiocytosis identified the coexpression of E-cadherin, a marker of LC differentiation, in 3/30 cats and could represent cats with LCH.^10^

## Case description

An 11-year-old spayed female domestic shorthair cat was referred with a 2-month history of ptyalism, progressive hyporexia and weight loss. Ptyalism was intermittent and had no association with feeding. Empirical treatment with cefovecin (8 mg/kg SC once [Convenia; Zoetis]), meloxicam (0.3 mg/kg PO q24h [Metacam; Boehringer Ingelheim]) and famotidine (1 mg/kg PO q24h [Famotidine; Teva]) was not beneficial. On presentation the cat was bright and responsive, weighed 2.5 kg (body condition score 2/9) and had lost 500 g weight over the previous month. Physical examination revealed a mild generalised muscle atrophy. Oral and thyroid examination, thoracic auscultation and abdominal palpation were normal. A 6% dehydration was estimated based on a mild loss of skin elasticity.

Gastrointestinal disease was suspected. Complete blood count and serum biochemistry identified mild erythrocytosis (haematocrit 0.55 l/l; reference interval [RI] 0.24– 0.45 l/l) and elevated creatine kinase (384 IU/l; RI 70–180 IU/l). In-house testing for feline immunodeficiency virus and feline leukaemia virus were negative. A gastrointestinal panel revealed mild hypercobalaminaemia and hypofolataemia (Table 1). Ammonia was measured to rule out hepatic encephalopathy as a cause of the ptyalism and was within the RI.

**Table 1:** Gastrointestinal panel

Test	Result	RI
fTLI (µg/l)	22.9	12–82
Cobalamin (pmol/l)	680	220–500
Folate (nmol/l)	17.7	19.0–37.0
fPL (µg/l)	0.5	0.0–3.5

RI = reference interval; fTLI = feline trypsin-like immunoreactivity; fPL = feline pancreatic lipase

The owner was offered a CT of the head and thorax of the cat to better assess for possible hidden dental/oral disease, and assess the oesophagus, respectively, followed by abdominal ultrasound and an upper gastrointestinal endoscopy to investigate oesophageal and gastrointestinal disease. Abdominal ultrasound was chosen over CT to better assess the gastrointestinal wall layering. The owner initially declined the thoracic CT owing to financial concerns, given the absence of respiratory signs and because the oesophagus would be assessed during endoscopy.

CT study of the head (Brivo CT385; GE Medical Systems) with contrast medium (Iopamidol, Niopam, 300 mg/ml; Bracco) and abdominal ultrasound showed no significant abnormalities. The upper gastrointestinal endoscopy (GIF-N230 flexible video-endoscope; Olympus Medical System) was suggestive of stenosis in the distal third of the oesophagus with a focal area of ulceration in the mucosal surface. The stomach and duodenum appeared macroscopically normal.

**Figure 1:**
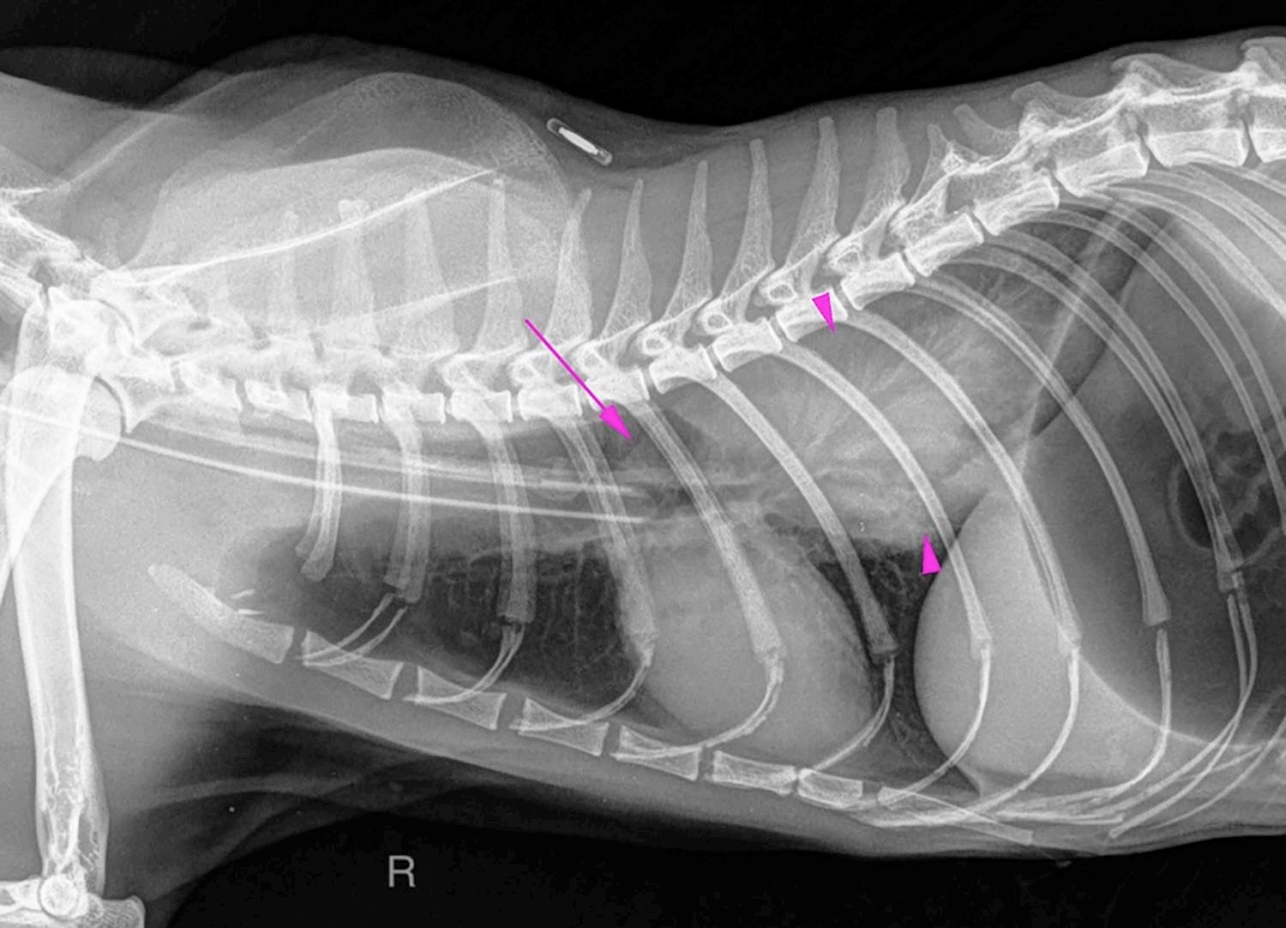
Right lateral radiograph of the thorax obtained after CT of the head and upper gastrointestinal tract endoscopy. There is a large soft tissue opacity mass lesion (pink arrowheads) in the caudodorsal thorax. Cranial to this mass, the lumen of the oesophagus is distended with gas (long pink arrow)

Given the suspicion of oesophageal stenosis, thoracic radiographs were obtained (Figure 1). They showed a large soft tissue opacity mass lesion in the caudodorsal thorax. Cranial to the mass the oesophagus was moderately distended with gas, but tapered abruptly at the level of the carina in the cranial margin of the mass. These findings were compatible either with a parietal oesophageal mass or other caudodorsal thoracic mass, caudal mediastinum or pulmonary in origin, causing dorsal compression of the oesophagus. At least three small (<3.5 mm) pulmonary nodules were detected in the cranioventral lung lobes. The gastrointestinal tract was distended with gas due to previous endoscopy and there was contrast medium in the urinary tract due to the previous CT.

**Figure 2:**
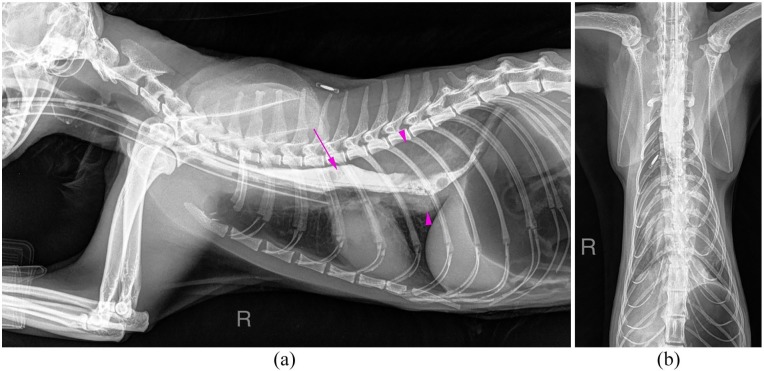
(a) An iodine oesophragram study was performed using an oesophageal tube (right lateral view). The lumen of the oesophagus (long pink arrow) containing positive contrast medium is surrounded dorsally and ventrally by the soft tissue opacity mass (pink arrowheads). (b) In the dorsoventral view, the mass lesion is in the midline, but it is not possible to differentiate if it is oesophageal or paraoesophageal

To further investigate the origin of the mass, an iodine oesophagram was performed using an oesophageal tube. Cranial to the mass, the oesophageal lumen was distended with diluted contrast medium (Iopamidol, Niopam, 300 mg/ml; Bracco). At the level of the mass, the lumen was slightly narrowed but patent. On the lateral views, the mass was seen surrounding the oesophageal lumen dorsally and ventrally (Figure 2a). The dorsoventral view confirmed that the mass was in the midline, but it was not possible to clearly define if the origin was oesophageal or paraoesophageal (Figure 2b).

**Figure 3:**
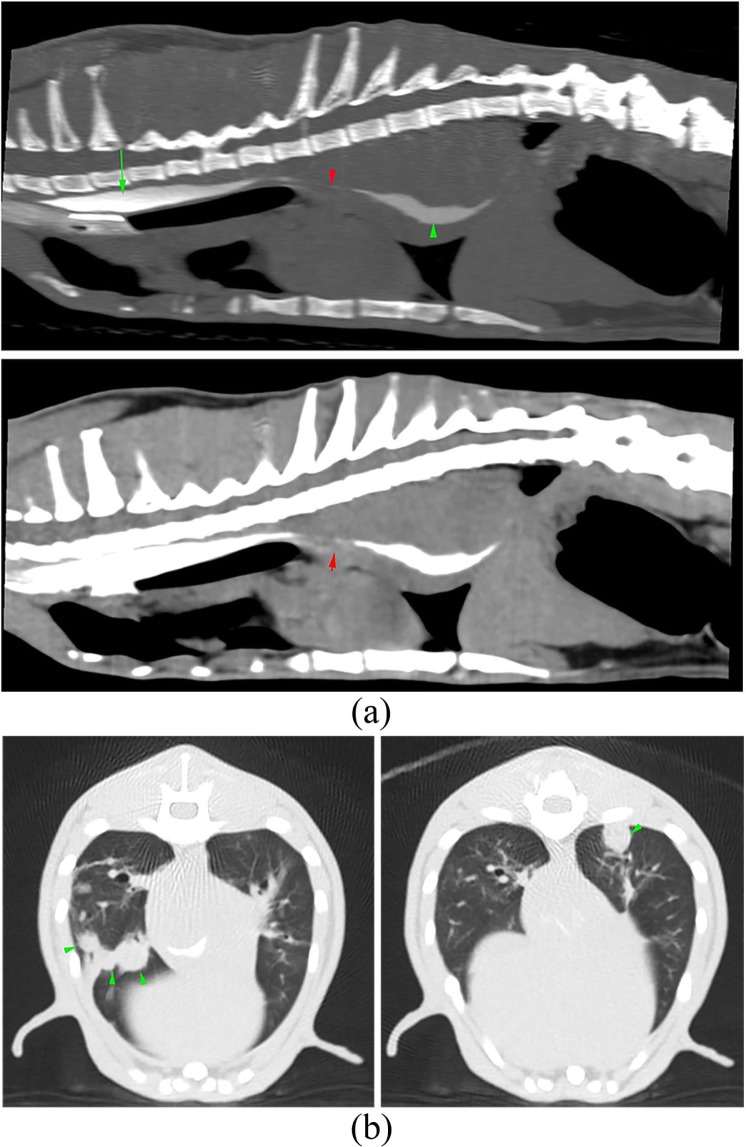
(a) Sagittal maximum intensity projection (MIP) reconstructions of the iodine oesophagram in bone (top) and soft tissue (bottom) algorithms. A large, well-defined parietal oesophageal mass is visible in the mid-caudal thorax. In the cranial mediastinum, the oesophageal lumen (long green arrow) is mildly to moderately distended with positive contrast medium. At the heart base, the lumen of the oesophagus is narrowed (short red arrow), and in the caudal thorax, the oesophageal lumen is ventrally displaced (green arrowhead). (b) Transverse MIP images of the thorax at the level of the caudal lung lobes in lung algorithm. The green arrowheads point to multiple pulmonary nodules (left) that coalesce in the right caudal lung lobe (right)

CT of the thorax was performed. The mediastinum was markedly widened by a well-defined, homogeneously attenuating parietal oesophageal mass (Figure 3a) extending from the level of thoracic vertebrae 7–13. In the cranial mediastinum, the lumen of the oesophagus was mildly to moderately distended with contrast medium. Dorsal to the heart base, a 1.5 cm length narrowing of the oesophageal lumen was detected. Caudal to it, the lumen of the oesophagus was narrow and ventrally displaced. Deviation and compression of the mainstem bronchi, owing to mass effect, and atelectasis of the lung adjacent to the mass were identified. Multiple round, well-defined, homogenously attenuating soft tissue nodules of various sizes were scattered throughout the lung parenchyma, some of them coalescing (Figure 3b). There were areas of peribronchial alveolar infiltrate on both sides of the mass, involving the left cranial and caudal lung lobes, and the right middle and caudal lung lobes, resulting in decreased lung volume.

**Figure 4:**
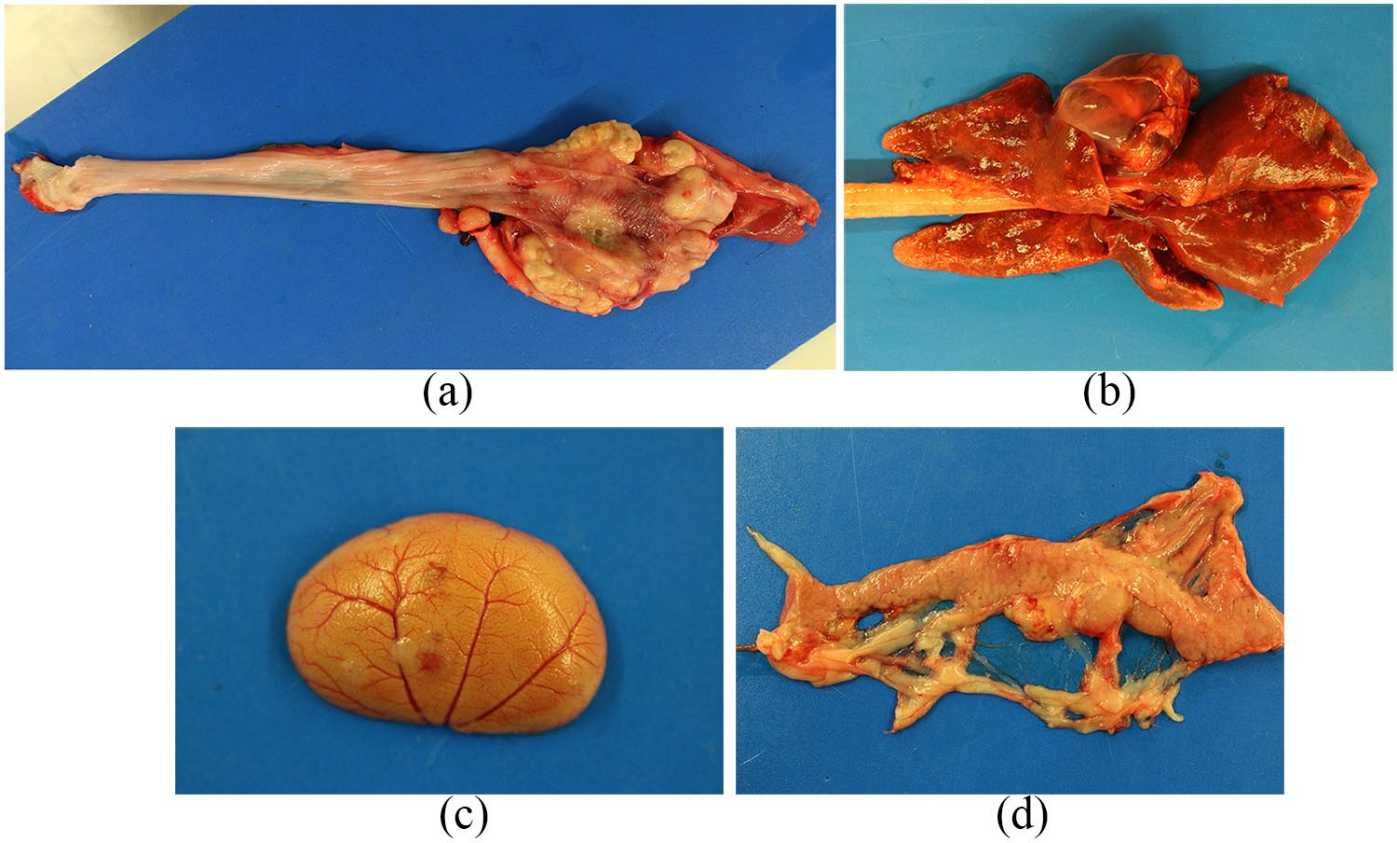
(a) An irregular, firm, white oesophageal mass in the distal third of the oesophagus. Ex situ, transected. (b) Lungs, (c) left kidney, (d) pancreas, ex situ. Multifocal to coalescing tan-coloured masses infiltrating the visceral parenchyma

Given the severity of clinical signs, diagnostic imaging findings and perceived guarded prognosis, the owner elected for euthanasia. On post-mortem examination the distal third of the oesophagus was severely enlarged due to the presence of a circumferential mass. This mass was firm and white, with evidence of mucosal ulceration (Figure 4a). Several nodular lesions measuring up to 5 mm were detected in the pulmonary parenchyma, with multifocal and random distribution (Figure 4b). Similar lesions were detected bilaterally in the kidneys and in the pancreas, with mild enlargement of the pancreatic lymph nodes (Figure 4c,d).

**Figure 5:**
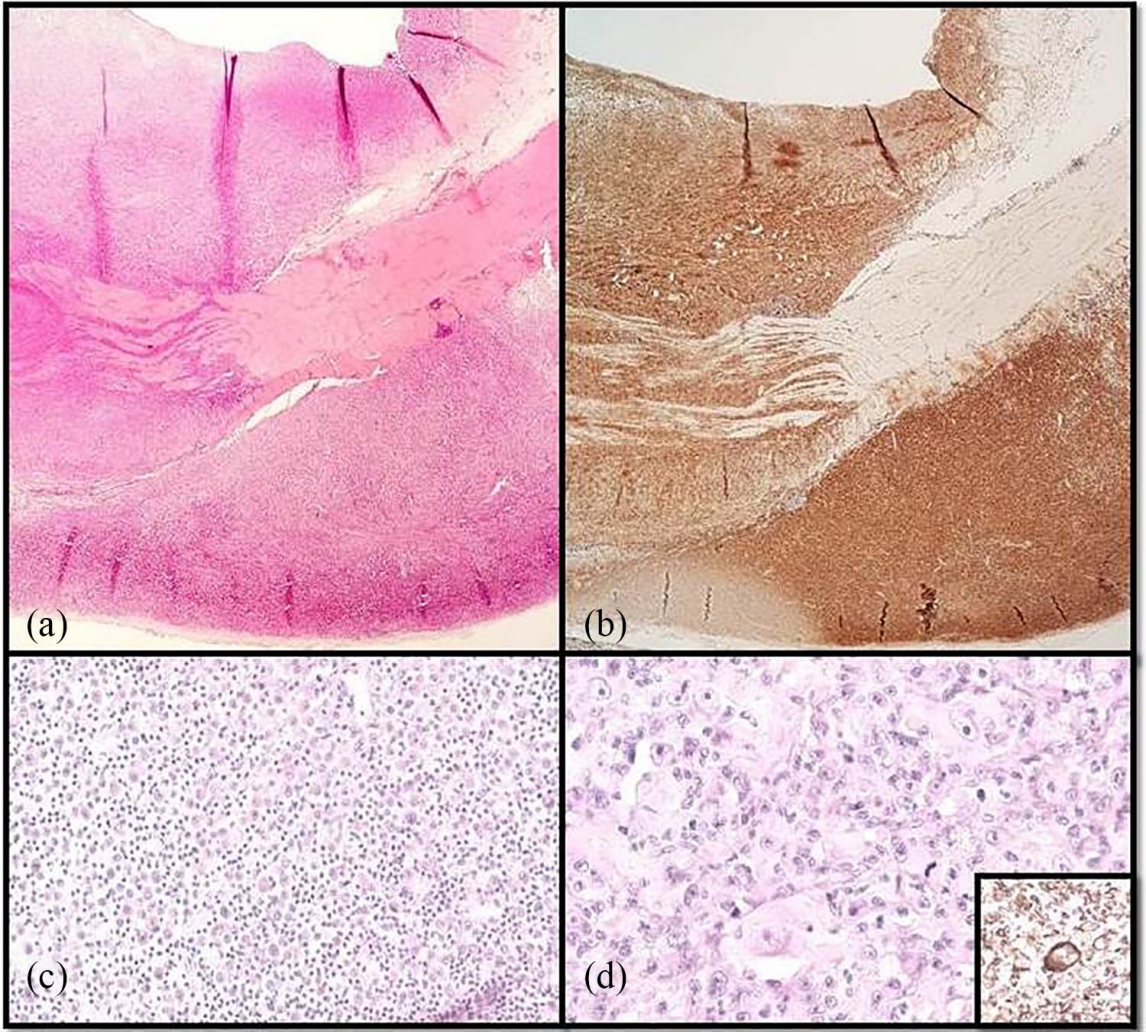
(a) The wall of the oesophagus is thickened owing to infiltration of atypical histiocytic cells; haematoxylin and eosin (× 20 magnification). (b) Infiltrating cells are diffusely and strongly positive for Iba-1; immunohistochemistry (× 20 magnification). (c) The round cells are admixed with neutrophils and small lymphocytes; haematoxylin and eosin (× 200 magnification). (d) Multifocally, histiocytes are characterised by cellular/nuclear atypia and mitotic activity; haematoxylin and eosin (× 400 magnification). Inset: atypical cells are strongly positive for Iba-1; immunohistochemistry (× 400 magnification)

Histopathological examination of the aforementioned organs revealed the presence of a population of round cells arranged in densely cellular sheets exhibiting distinct cell borders, and a moderate amount of eosinophilic cytoplasm with one oval, prominent, vesicular nucleus, which was frequently indented. Anisokaryosis and anisocytosis were moderate to severe. There were three mitoses per high-power field. High numbers of neutrophils and small lymphocytes were frequently associated with proliferating round cells. Immunohistochemical investigation was performed and the round cells showed strong expression for CD18 (FE3.9F2; UC Davis), Iba-1 (polyclonal; Wako) and vimentin (monoclonal; Dako), and were negative for chromogranin A (polyclonal; Dako), CD20 (monoclonal; Dako), CD3 (monoclonal; Dako), cytokeratin (monoclonal; Dako) and synaptophysin (polyclonal; Dako) (Figure 5).

**Figure 6:**
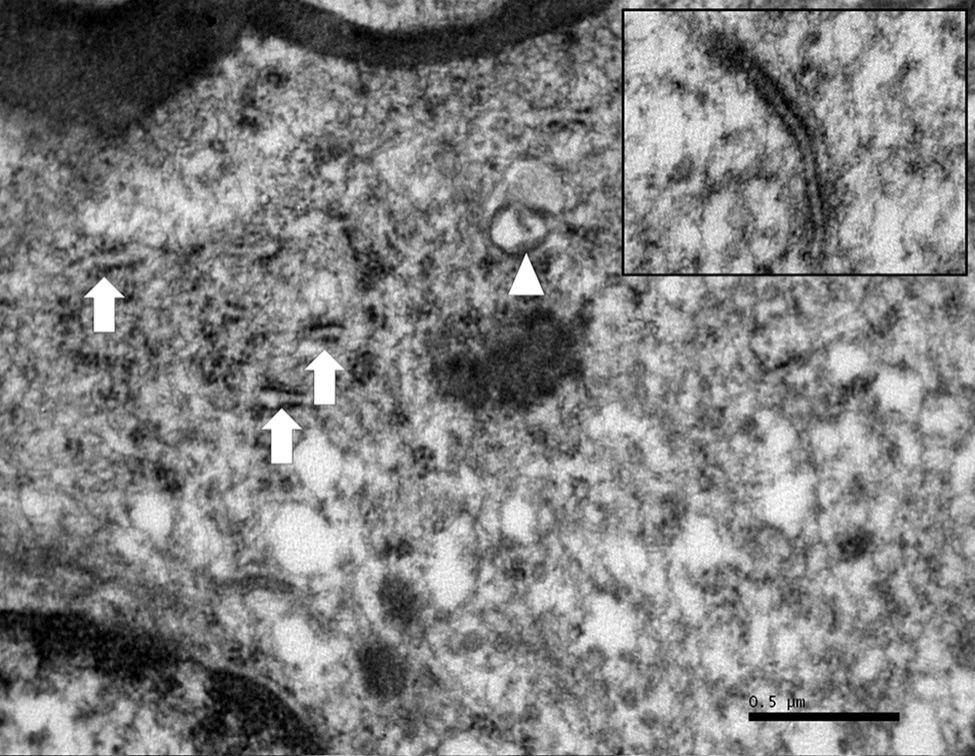
Transmission electron microscopy. Intracytoplasmic structures consistent with Birbeck granules (arrows) occasionally form bulbous structures (arrowhead). Inset: high-power magnification of a single granule with evidence of bilaminar structures with an internal zipper-like pattern

Selected formalin-fixed paraffin-embedded tissue samples were submitted for transmission electron microscopy (TEM). Ultrastructural morphology was suboptimal owing to the formalin fixation and paraffin embedding; however, diagnostic features were recognised. Atypical histiocytic cells in multiple locations revealed evidence of intracytoplasmic electron-dense bilaminar structures characterised with an internal zipper-like pattern of striations consistent with BGs of LC origin. Occasionally, terminal bulbous structures were observed (tennis racquet morphology) (Figure 6). These findings were consistent with a final diagnosis of disseminated LCH.

## Discussion

Gastrointestinal involvement in LCH is rare in humans and oesophageal involvement is poorly documented.^11,12^ Oesophageal involvement in disseminated LCH in cats has not been previously described. Reported cases have presented primarily with respiratory complaints, lethargy, anorexia or weight loss.^5,9^ In our case hyporexia, weight loss and ptyalism were the presenting clinical signs. While ptyalism is a common clinical sign of oesophageal disease, pseudoptyalism from an involuntary disruption of the swallowing mechanism is also a likely consequence of mechanical obstruction from the oesophageal mass.^13^

There are no typical clinicopathological findings associated with LCH, and no known association with retroviruses.^5,9^ In the present case, the mild erythrocytosis was considered secondary to dehydration and the elevated creatine kinase due to oesophageal damage. Hypofolataemia was most consistent with decreased intake. Hypercobalaminaemia has been associated with solid neoplasms in cats, and seems the most likely cause in the absence of supplementation.^14^

Thoracic radiographs of pulmonary LCH report mixed pulmonary patterns that are bronchointerstitial, alveolar or interstitial, with or without nodular opacification.^5,9^ In this case, pulmonary nodules were detected within the cranioventral lung lobes. Peribronchial alveolar infiltrates on both sides of the mass in combination with reduced lung volume was interpreted as lung atelectasis. The anatomical origin of the caudodorsal thoracic mass could not be defined based on survey radiographs alone. It might have been originating from the caudal mediastinum or lung parenchyma. The iodine oesophagogram demonstrated the relationship between the mass and the oesophageal lumen that was surrounded dorsally and ventrally by the mass. This might have been explained by a parietal oesophageal mass or a paraoesophageal mass. As oesophageal masses are rare in cats, the decision was made to perform CT of the thorax to define the anatomical origin of the lesion. CT was the definitive imaging modality to define the anatomical origin of the mass and allowed a better characterisation of the size and distribution of the pulmonary nodules.

Infiltration of the affected organs by multifocal-to-coalescing nodular masses in the present case are similar to the characteristic feature described in pulmonary LCH.^5,9^ Although lesional histiocytes exhibited similar cellular and nuclear descriptions of pleomorphism, there are differences between the present case and reports of pulmonary LCH. Pulmonary LCH has been characterised by cohesive infiltration of histiocytic cells, which obliterate terminal airways and extend to the pleural surface.^5,9^ Accompanying inflammatory cells are dominated by lymphocytes, with fewer plasma cells and occasional macrophages.^9^ In our case a predilection for terminal airways was not observed and neutrophilic infiltration contributed significantly to the proportion of accompanying inflammatory cells.

Immunohistochemical profiles of histiocytic cells are a cornerstone in the diagnosis of LCH.^15^ Expression of Iba-1 has been determined as a useful marker for histiocytic proliferative, neoplastic and inflammatory disorders in cats, although it is unable to differentiate between macrophage and dendritic antigen presenting cells.^16^ In addition, the use of CD18 expressed on all leukocytes, as a histiocytic marker, is dependent upon exclusion of lymphocyte differentiation as demonstrated in this case.^9^ In keeping with previous reports of pulmonary LCH, lesional histiocytes expressed vimentin, Iba-1 and leukointegrin CD18, alongside negative immunolabelling of cytokeratin and lymphoid differentiation antigens CD3 and CD20.^5,9^ Expression of CD1a and adhesion molecule E-cadherin was not assessable; however, a diagnosis was achieved by the identification of BGs on TEM, which are a hallmark of histiocytes of LC origin.^17,18^

Financial restraints were a limitation and influenced the diagnostic investigations performed in this case. Initially, performing an oral examination and thoracic radiographs after anaesthesia would have given relevant information to guide the case, then followed by CT of the head and thorax to rule out hidden oral disease and better characterise the thoracic changes seen on radiography, respectively. Endoscopic biopsies of the affected oesophagus were obtained but not analysed because a full post-mortem examination was performed. Given that the neoplastic cells involved all layers of the affected oesophagus these biopsies would likely be suitable to reach a diagnosis in an ante-mortem setting.

## Conclusions

We present a novel case of disseminated LCH with oesophageal involvement in a cat. Feline LCH should be included in the differential diagnosis list of ptyalism and when there is a clinical index of suspicion of oesophageal disease. Ante-mortem diagnosis and management of LCH in the cat remains undescribed and requires future studies.

## References

[1] Clifford CA Skorupski KA Moore PF Histiocytic diseases. In: Withrow SJ Vail DN Page RL (eds). Small animal clinical oncology. 5th ed. St Louis, MO: Elsevier Saunders, 2013, pp 706–713.

[2] Larregina AT Morelli AE Dermal-resident CD14+ cells differentiate into Langerhans cells. Nat Rev Immunol 2001; 2: 1151–1158. 10.1038/ni731 11702065

[3] Merad M Ginhoux F Collin M Origin homeostasis and function of Langerhans cells and other langerin-expressing dendritic cells. Nat Rev Immunol 2008; 8: 935–947. 19029989 10.1038/nri2455

[4] Moore PF A review of histiocytic disease of dogs and cats. J Vet Pathol 2014; 51: 167–184. 10.1177/0300985813510413 24395976

[5] Bush MDM Reilley MC Luff JA, et al Feline pulmonary Langerhans cell histiocytosis with multiorgan involvement. Vet Pathol 2008; 45: 816–824. 18984784 10.1354/vp.45-6-816

[6] Meyer W Hornickel I Schoennagel B A note on Langerhans cells in the oesophagus epithelium of domesticated mammals. Anat Histol Embryol 2009; 39: 160–166. 10.1111/j.1439-0264.2009.00990.x 20085569

[7] Steinman RM Inaba K Turley S, et al Antigen capture, processing, and presentation by dendritic cells: recent cell biological studies. Hum Immunol 1999; 60: 562–567. 10426272 10.1016/s0198-8859(99)00030-0

[8] Histiocyte Society. Evaluation and treatment guidelines. https://histiocytesociety.org/document.doc?ID=290 (2009, accessed April 2019).

[9] Argenta FF de Britto CF Pereira PR, et al Pulmonary Langerhans cell histiocytosis in cats and a literature review of feline histiocytic diseases. J Feline Med Surg. Epub ahead of print 12 4 2019 DOI: 10.1177/1098612X19842384. PMC10814658 30977699

[10] Affolter VK Moore PF Feline progressive histiocytosis. Vet Pathol 2006; 43: 646–655. 16966441 10.1354/vp.43-5-646

[11] Singhi AD Montgomery EA Gastrointestinal tract Langerhans cell histiocytosis: a clinicopathologic study of 12 patients. Am J Surg Pathol 2011; 35: 305–310. 21263252 10.1097/PAS.0b013e31820654e4

[12] Behdad A Owens SR Langerhans cell histiocytosis involving the gastrointestinal tract. Arch Pathol Lab Med 2014; 138: 1350–1352. 25268199 10.5858/arpa.2014-0290-CC

[13] Heinze C Niemiec BA Pytalism and halitosis. In: Ettinger SJ Feldman EC Côté E (eds). Textbook of veterinary internal medicine. 8th ed. St Louis, MO: Elsevier Saunders, 2015, pp 146–151.

[14] Trehy MR German AJ Silvestrini P, et al Hypercobalaminaemia is associated with hepatic and neoplastic disease in cats: a cross sectional study. BMC Vet Res 2014; 10: 175. DOI: 10.1186/s12917-014-0175-X. 25103858 10.1186/s12917-014-0175-x PMC4236818

[15] Jaffe R Weiss LM Facchetti F Tumours derived from Langerhans cells. In: Swerdlow SH Campo E Harris NL, et al (eds). WHO classification of tumors of the haematopoietic and lymphoid tissues. 4th ed. Lyon: IARC, 2008, pp 358–360.

[16] Pierezan F Mansell J Ambrus A, et al Immunohistochemical expression of ionized calcium binding adapter molecule 1 in cutaneous histiocytic proliferative, neoplastic and inflammatory disorders of dogs and cats. J Comp Pathol 2014; 151: 347–351. 25172051 10.1016/j.jcpa.2014.07.003

[17] Woo JC Moore PF A feline homologue od CD1 is defined using a feline-specific monoclonal antibody. Tissue Antigens 1997; 49: 244–251. 9098931 10.1111/j.1399-0039.1997.tb02745.x

[18] Pinto da Cunha N Ghisleni G Scarampella F, et al Cytologic and immunocytochemical characterization of feline progressive histiocytosis. Vet Clin Pathol 2014; 43: 428–436. 24820657 10.1111/vcp.12152

